# Investigation of Molecular Mechanism of Chronic Pain in the Anterior Cingulate Cortex Using Genetically Engineered Mice

**DOI:** 10.2174/138920210790217990

**Published:** 2010-03

**Authors:** Susan S. Kim, Giannina Descalzi, Min Zhuo

**Affiliations:** 1 Department of Physiology, Faculty of Medicine, University of Toronto, Centre for the Study of Pain, 1 King’s College Circle, Toronto, ON, Canada; 2 Department of Brain and Cognitive Sciences, Seoul National University, Seoul 151-746, Korea

## Abstract

Recent advances into the understanding of molecular mechanism of chronic pain have been largely developed through the use of genetic manipulations. This is in part due to the scarcity of selective pharmacological tools, which can be readily solved by creating knockout or transgenic mice. By identifying new genes that are of import, our efforts can then be aimed at studying relevant signaling pathways, and combination of pharmacological manipulations with genetic models can be used to further examine the specific mechanisms involved in chronic pain. In this review, we will examine the genetic models that are currently in use to study chronic pain in the anterior cingulate cortex: knockout mice; transgenic mice; and the strength of combining pharmacology with these genetic models.

## INTRODUCTION

Pain is an essential sensory mechanism that warns us of imminent tissue damage. However, clinical problems associated with pain occur when pain thresholds are dramatically altered. Pain is divided into two major categories: Acute or physiological pain and chronic or pathological pain [1,2]. Unlike physiological pain, which helps us to determine potentially dangerous stimuli and avoid it, making it essential for survival, pathological pain has no benefit and only occurs after insult (e.g., tissue or nerve injury or disease) [3,4]. In addition to spontaneous occurrences of pain, there are two common pathological conditions that develop after tissue or nerve injury: Allodynia, where the pain threshold has been reduced, allowing non-noxious stimuli that normally do not cause pain to induce pain, and hyperalgesia, in which there is an enhanced response to noxious stimuli [2].

Recent studies using animal models as well as human brain imaging techniques have consistently revealed that chronic pain is likely due to sensitization along the somatosensory pathways, including long-lasting changes at peripheral tissue, spinal cord, and cortex [2]. Furthermore, descending biphasic modulatory systems that control the amount of pain information reaching the brain also undergo plastic changes [5]. Still, much about the molecular mechanisms of chronic pain remain to be explored. Integrative experimental approaches including electrophysiological, pharmacological, biochemical, anatomical, and behavioral methods have proven to be useful for investigating the molecular mechanisms of chronic pain. In this review, we will focus on the use of genetically manipulated mice in studying synaptic and molecular mechanisms of chronic pain in the anterior cingulate cortex (ACC), an area known to mediate the emotional component of pain. We believe that the use of genetically manipulated mice offers not only complete inhibition of the activity of target proteins than traditional pharmacological inhibitors, but better selectivity as well. Furthermore, the findings from genetic studies will help in the development of novel and effective therapeutic agents to combat chronic pain.

## MOUSE MODELS FOR INVESTIGATING CHRONIC PAIN

The mechanism of nociception is usually studied in rodents as the various stimuli used mimic clinical pain in humans. Additionally, mice and humans share approximately 99% of their genes [6], which allows mice to be used as models of human physiology. A wide variety of chronic pain assays have been developed for the mouse, each examining different pain modalities. Inflammatory pain, which results from tissue damage and is reversible when the underlying cause has been rectified, is most commonly studied using the formalin test and injections of complete Freund’s adjuvant (CFA). The formalin test, a common test of tissue injury and inflammation on a timescale of hours, is used to model “acute” inflammation. Formalin is injected into the dorsum of the hindpaw, then spontaneous responses to the injection are measured [7,8]. Formalin-induced behavioral responses are typically categorized into two distinct phases: Phase one (0-10 min) and phase two (10-55 min) [8], although we have previously shown that mice continue to exhibit behavioral responses during the 55-120 min period, a “phase three” [9]. Formalin-induced behavioral responses also depend on NMDA receptors at different levels of the brain [10]. CFA, on the other hand, is a water-in-oil emulsion that contains heat killed *Mycobacterium tuberculosis* cell wall components. Much like formalin, CFA is injected into the dorsum of the hindpaw, although its effects can last for days. Following CFA injection, allodynia is typically observed on day 1 and 3 after injection by applying von Frey filament to the dorsum of the hindpaw to measure the withdrawal responses.

Neuropathic pain results from damage to components of the nervous system, such as primary afferent nerves, spinal cord, and cortical regions [11]. As the onset of neuropathic pain may be delayed after a nerve injury, pain may still be present even after healing is complete [4,11]. Moreover, neuropathic pain commonly occurs as a secondary symptom in diseases like diabetes and cancer, and may also occur with treatments, such as chemotherapeutics or cytotoxic drugs [11]. Furthermore, due to the dynamic nature of the pain system, the signs and symptoms of neuropathic pain change over time, and injury to peripheral nerve causes functional and biochemical change to not only at the site of injury, but also to other parts of affected nerve and eventually to higher order neurons in the spinal cord and brain [4]. A number of animal models have been reported to simulate human peripheral neuropathic pain conditions. The peripheral nerve injury models have been the most extensively studied, with the exact methods differing in the location and form of injury, including transaction and ligation [12]. Partial injury to the sciatic nerve is most commonly used to induce neuropathic pain behavior, including loose ligation of the sciatic nerve, developed by Bennett and Xie [13], as well as spinal L5/L6 nerve ligation, developed by Kim and Chung [14]. Recently, we have discovered a novel mouse model of neuropathic pain, a simple and less invasive procedure where the common peroneal nerve is ligated, leaving motor function intact [15]. The presence of neuropathic pain is mainly assessed by hyperalgesia to thermal and mechanical stimuli and allodynia to cold and tactile stimuli [12,14,16-18].

In addition to inflammatory pain and neuropathic pain, a wide variety of other pain assays have been developed to assess physiological and pathological pain in mice, such as thermal nociception and visceral pain. List of the most used models have been provided below (see Table **1**).

## DELETION OF SIGNALING GENES

The availability of genetic mouse models has revolutionized biomedical research, and alterations to classical gene modification techniques have made possible the generation of conditional, inducible, and multi-gene knockout mouse mutants. These techniques deliver a more detailed and informative look into the *in vivo* functions and endogenous expression patterns of individual genes.

The basic premise behind the development of a knockout mouse is to replace the normal functioning gene with one that is nonfunctional (i.e., a null mutation) using homologous recombination. By comparing the phenotypes of these knockout mice to those of wild-type mice, the function of the targeted gene could then be deduced. If phenotypic differences exist between the knockout mice and their wild-type counterparts, it strongly suggests that the gene is responsible for or regulates that particular function. However, knockout technology has its limitations. Many knockout mice are embryonically lethal due to developmental defects, and some genes may serve a different function in adults than in developing embryos [19]. In such cases, conditional and inducible gene knockout technology can be used. Unlike the conventional knockout technology where the gene of interest is completely absent from all cells, conditional knockouts by contrast inactivate the gene of interest in a tissue-specific manner, allowing greater control over gene expression. An inducible knockout enables the researcher to activate or inactivate the gene of interest at specific developmental time points, using antibiotic-controlled transcriptional activation, so that gene expression can be reversibly induced in the presence or absence of antibiotics, such as tetracycline [19].

There are several different ways to make conditional knockout models, but the most widely used is the Cre-*lox*P recombinase system, where the target gene is modified through Cre action [20]. Cre expression can also be controlled temporally, by introducing an element into the promoter that requires a ligand, such as a drug, for induction [21].

## REGIONAL INHIBITION OF PROTEIN FUNCTIONS

### siRNA

In the past few years, RNA interference (RNAi) has revolutionized studies to determine the role of a gene and has become the predominant means of assessing loss of gene function in most organisms [22]. RNAi provides a rapid and easy means of depleting mRNAs by interfering with the expression of a specific gene [22]. Small interfering RNA (siRNA), sometimes known as short interfering RNA or silencing RNA, is generally 20-25 basepairs long, and can be artificially introduced into cells by various transfection methods to bring about the specific knockdown of a gene of interest [23]. Any gene with a known sequence can be targeted based on sequence complementarity with an appropriately tailored siRNA, making it an important tool for gene function studies.

The efficacy of siRNA depends on multiple factors, including GC content and the thermodynamic stability of the duplex at the 5’ antisense end [24]. Several types of vectors for siRNA delivery have been developed to not only protect siRNAs from degradation but also for site-specific delivery and to enhance its cellular uptake. While non-viral vectors have the advantages of low toxicity, ease of synthesis and low immune response [25], viral vectors have the advantage of delivering siRNAs to neurons as well as glial cells, making it the most commonly used carriers for gene transfer. However, targeting specific cell types still remain a challenge. The advantage of efficient, economical knockdown offered by RNAi and the large amount of data provided makes it the current technology of choice, although overcoming the delivery obstacle for *in vivo* studies remains.

### Electroporation

Much like RNAi, the ability to transfer any gene of interest to a specific brain region has been a much sought-after technique. Several approaches have been used to target gene transfer to neurons *in vivo*, including the use of adenoviral-based vectors [26-28] and liposomal-plasmid complexes [29,30]. Electroporation, a technique where brief electrical pulses are applied to tissue to temporarily increase cellular permeability to DNA, has been used to enhance gene delivery into cells of adult organs, as well as neurons in the brains of embryonic mice [31]. Electroporation enables selective delivery of a gene to specific regions of the central nervous system (CNS), but unlike the transgenic approach, electroporation provides a method for unilateral gene transfer, allowing overexpression of a gene within a highly specific brain region [31]. Additionally, electroporation provides a way to express a gene at any time during the development of normal or pathophysiological processes, allowing for investigations of the role of signaling proteins in numerous brain functions, and provides a method to rescue behavioral defects observed in genetically altered mice with fewer toxic effects when compared to other methods of gene transfer [31]. We have previously developed a microelectroporation method and selectively expressed both green fluorescent protein (GFP) and mutant calmodulin (CaM) in the ACC of adult rodents, the first instance where electroporation was used to deliver genes into the adult mammalian brain [31]. And recently, we have successfully combined siRNA and electroporation to reduce the level of NR2B expression in the ACC of mice [32].

## GENETIC MANIPULATIONS IN INVESTIGATING CHRONIC PAIN IN THE ACC

Elucidating the function of particular neurotransmitters and their receptors has traditionally relied upon pharmacological approaches using compounds that act as specific agonists or antagonists at defined receptors on the pre- and postsynaptic membrane [33]. Unfortunately, selective antagonists of ion channels are limited, and lesion studies and local chemical injection experiments to dissociate behavioral responses to acute noxious stimuli from long-term sensitization to the injury have failed, as these manipulations alone significantly affect behavioral responses to acute stimuli [7]. Therefore, researchers have turned to knockout and transgenic mice to investigate pain pathways.

### The Use of AC1 and AC8 Gene Knockout Mice

**Fig. (1). Identifying a plasticity-selective gene for treating chronic pain.**
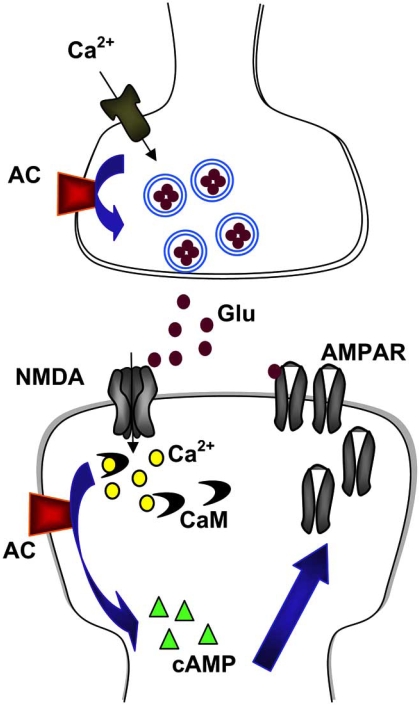
Inflammatory or nerve injuries cause both presynaptic and postsynaptic changes in the ACC synapses. Activity triggers the release of excitatory neurotransmitter glutamate, and activation of NMDAR leads to an increase in postsynaptic Ca^2+^. Ca^2+^ binds to CaM and leads to activation of calciumstimulated AC1 and Ca^2+^/CaM-dependent protein kinases, resulting in possible postsynaptic trafficking of AMPA GluR1 receptors as well as increasing AMPAR sensitivity to glutamate. Enhanced release of glutamate as well as postsynaptic changes in AMPAR-mediated responses contributes to enhanced noxious sensory information within the brain, and may be a possible cellular mechanism for chronic pain.

cAMP-related signaling pathway has previously been shown to contribute to the behavioral responses to tissue injury and inflammation in the periphery and the spinal cord [34-37], but few studies have addressed the potential roles of forebrain cAMP in such changes. AC1 and AC8, two CaM-stimulated adenylyl cyclases (AC) in the CNS, couple NMDA receptor-induced cytosolic Ca^2+^ increases to cAMP signaling pathways [38,39]. To examine the roles of AC1 and AC8 in behavioral sensitization within the ACC, an area mediating the emotional component of pain, we used AC1 and AC8 knockout mice as well as AC1&8 double knockout (DKO) mice to determine the roles of these ACs in chronic pain [7] (Fig. **1**). Behavioral nociceptive responses to subcutaneous formalin injection or nerve injury were significantly reduced in mice lacking AC1 or AC8, and more profoundly compromised in AC1&8 DKO mice. Allodynia was significantly reduced in AC1 KO mice and absent in AC1&8 DKO mice, but not in AC8 KO mice, indicating that ablation of AC8 alone is not sufficient to affect allodynia. We found that AC1 and AC8 were both expressed at high levels in two pain-related forebrain areas, the ACC and the insular cortex. Next, in order to determine whether the lack of allodynia was due to an acute requirement for AC1 and AC8 or the developmental changes in cortical function that occurred due to chronic deficiency of these isoforms, we attempted an acute behavioral rescue. Forskolin, a nonselective AC activator, was microinjected into the ACC of mice 1 day following CFA injection to activate ACs, and we found that AC1&8 DKO mice were hypersensitive to the non-noxious stimulus. Therefore, AC1 and AC8 are important for behavioral nociceptive responses to nerve injury and inflammation, and the results suggest that AC1 and AC8 play important roles in the processing of nociceptive information in the ACC.

### Chemically-Engineered CaMKII Transgenic Mice

**Fig. (2). Two examples of combining chemical and genetic approaches.**
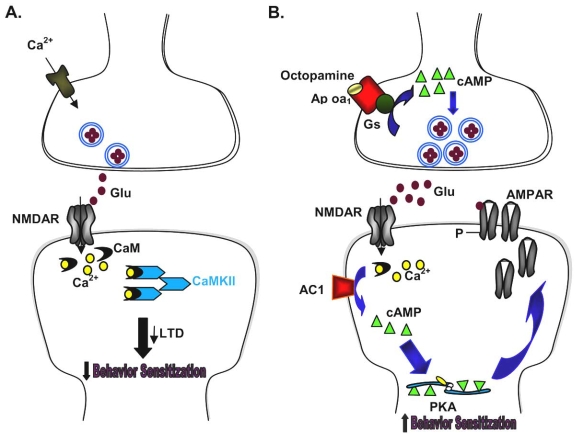
**A**. Inflammatory pain triggers the release of excitatory neurotransmitter glutamate, and activation of NMDAR leads to an increase in postsynaptic Ca^2+^. Ca^2+^ binds to CaM and leads to activation of Ca^2+^/CaM-dependent protein kinases such as CaMKII. Overexpression of CaMKII leads to reduction in LTD as well as allodynia and hyperalgesia, suggesting that the observed reduction in behavioral sensitization may be due to the loss of LTD. **B**. Activation of presynaptic Ap oa_1_ by octopamine leads to the production of cAMP. The cAMP-related signaling pathways then facilitate the release of glutamate, which in turn acts on NMDAR and AMPAR. This triggers the downstream signaling pathways, leading to increased behavioral sensitization.

Ca^2+^/CaM-dependent kinase II (CaMKII) is known to be a key molecule involved in regulating tissue-injury evoked persistent pain [40]. To determine the role of forebrain CaMKII in the development of persistent pain, we used CaMKII F89G transgenic mice to spatially and temporally restrict the expression of CaMKII to the forebrain areas [41,42]. These mice were developed using the conditional protein knockout/manipulation technique to achieve monospecific inhibition of a single kinase in one region of the mouse brain [41]. This method combined high temporal resolution of chemical inhibition with molecular and spatial specificity of genetic techniques [41]. We found that overexpressing CaMKII in the forebrain caused selective changes in synaptic LTD without causing any obvious anatomical abnormality or changes in neuronal excitability. Additionally, enhanced CaMKII activity in the forebrain resulted in significant reduction in mechanical allodynia 1 and 3 days after CFA injection into the dorsum of a single hindpaw, as well as reduction in thermal hyperalgesia. CaMKII mice were then treated with the inhibitor 1NM-PP1 to turn off the overexpressed CaMKII activity. We found that the inhibitor treatment completely blocked the effects of CaMKII overexpression on behavioral allodynia and hyperalgesia in transgenic mice, and synaptic transmission was rescued as well. The results suggest that the enhanced forebrain CaMKII activity reduced behavioral sensitization to peripheral injury, and that the loss of LTD in CaMKII transgenic mice may be responsible for the reduction of nociceptive responses after tissue injury (Fig. **2A**).

### Octopamine Mice

To investigate the synaptic mechanisms related to inflammatory pain, we used transgenic mice with heterologously expressed receptors impacting the cAMP pathway [43]. The mice heterologously expressed an *Aplysia* octopamine receptor (Ap oa_1_) [44], a G protein-coupled receptor selectively activating the cAMP pathway after the binding of its natural ligand, octopamine [45]. There were at least two advantages in using these transgenic mice: First, as Ap oa_1_ receptor expression is driven by CaMKIIα promoter, the receptor is selectively expressed in forebrain neurons, allowing the cAMP pathway coupled to Ap oa_1_ receptor to be spatially activated; and second, that since the receptor is not activated endogenously, the exogenous application of octopamine is the only way of activating Ap oa_1_ receptor and the associated cAMP pathway.

Octopamine is a trace amine and its endogenous level is far below those of classical neurotransmitters. Therefore, we selectively activated cAMP pathway by microinjecting octopamine into the ACC then tested behavioral responses to the formalin test [43]. We found enhancement in the intermediate inflammatory phase but not in the acute phase, indicating that the enhancement is selective for late phase responses. And activation of Ap oa_1_ receptors in the ACC enhanced presynaptic glutamate release but didn’t modulate postsynaptic function, suggesting that these heterologous receptors are expressed in presynaptic terminals modulating vesicle release, and that increased presynaptic glutamate release in the ACC is sufficient for enhancing chronic pain (Fig. **2B**).

### Forebrain NR2B Overexpressed Mice

**Fig. (3). Identifying a cognitive gene in chronic pain.**
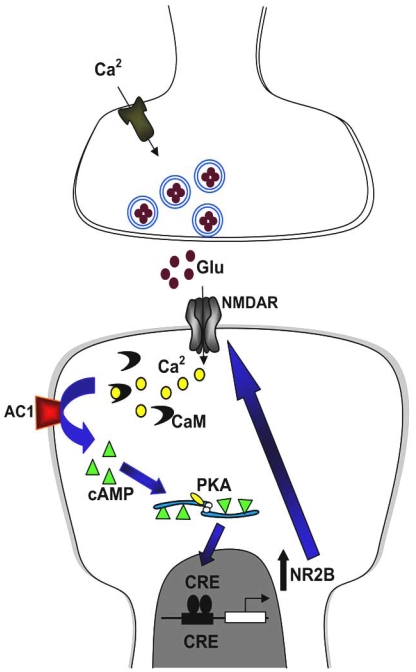
Peripheral injury such as tissue inflammation or nerve injury leads to a burst of abnormal activity, and subsequently activates postsynaptic NMDAR. This triggers Ca^2+^ influx, leading to activation of Ca^2+^/CaM-dependent pathways, including AC1. AC1 activation leads to the generation of cAMP, which activates PKA. PKA then translocates to the nucleus and phosphorylates CREB. Postsynaptic synthesis of NMDA NR2B is then increased, and the new NR2B subunits are added to postsynaptic NMDAR. This may further enhance neuronal excitability and contribute to chronic pain.

Functional NMDA receptors contain heteromeric combinations of the NR1 subunit plus one or more of NR2A-D [46], of which the NR2A and NR2B subunits are the major NR2 subtypes found in forebrain structures [47]. Using transgenic mice with forebrain-targeted overexpression of the NMDA receptor subunit NR2B, we examined their response to tissue injury and inflammation [8]. We found that transgenic mice exhibited prominent NR2B expression and enhanced NMDA receptor-mediated synaptic responses in two pain-related forebrain areas, the ACC and insular cortex. Additionally, these mice exhibited enhanced responsiveness to peripheral injection of two inflammatory stimuli: Formalin, where the enhancement was observed in the last phase, and CFA, where at day 1 and 3 after CFA injection, mechanical allodynia was significantly enhanced in the transgenic mice. We then showed that the NR2B receptor undergoes prolonged upregulation in the ACC neurons after tissue inflammation, with enhanced NMDA receptor-mediated responses [48]. Inhibition of NR2B receptors by administering selective NR2B receptor antagonists (Ro 25-6981 and Ro 63-1908) systematically as well as by bilateral microinjection into the ACC inhibited inflammation-related allodynia, suggesting that this increase contributes to chronic pain by enhancing the neuronal activity of the ACC (Fig. **3**).

### CaMKIV Gene Knockout Mice

**Fig. (4). CaMKIV distinguishes fear memory and chronic pain.**
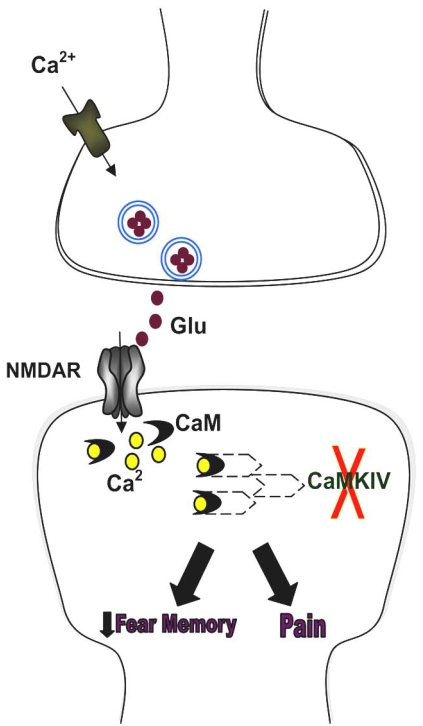
Inflammatory or nerve injuries trigger the release of excitatory neurotransmitter glutamate, and activation of NMDAR leads to an increase in postsynaptic Ca^2+^. Ca^2+^ binds to CaM and leads to activation of Ca^2+^/CaM-dependent protein kinases, such as CaMKIV. In the absence of CaMKIV, fear memory was significantly reduced but behavioral responses to painful stimuli remained unchanged, indicating that fear memory and pain may be dissociated at the molecular level.

There has been some debate over whether increased behavioral responses seen in “smart” mice on chronic pain tests genuinely reflect the severity of pain rather than the mice being better learners. In order to address this concern, we conducted fear memory as well as pain tests on CaMKIV knockout mice. CaMKIV, like CaMKII, is a key Ca^2+^/calmodulin-dependent kinase, and functions as a transcriptional activator [49-51]. We found that fear memory was significantly reduced in CaMKIV knockout mice, but behavioral responses to an acute noxious stimulus or prolonged injury remained comparable to wild-type mice [52]. The results indicate that CaMKIV is preferentially involved in fear memory induced by a noxious shock but not in behavioral responses to acute noxious stimuli or tissue inflammation, and that the two can be disassociated at the molecular level (Fig. **4**). Therefore, although many signaling pathways may contribute to normal physiological functions as well as to distorted pathological conditions, it is possible to identify signaling molecules that preferentially contribute to one but not the other.

## SUMMARY AND FUTURE DIRECTIONS

Recent advances in genetic manipulations have transformed physiology and medicine by allowing for a direct investigation into the function of specific genes, significantly improving our knowledge of the pain pathway. Previous studies have indicated that in the ACC, Ca^2+^-stimulated cAMP pathways are necessary for chronic pain behaviours, and that selective elevation of cAMP is sufficient to enhance chronic pain. As well, NMDA receptors and their related signaling pathways in the forebrain have been implicated in injury-related behavioral sensitization [8,31,53]. Consistently, behavioral sensitization after tissue injury appears to be regulated or modulated by neuronal activity within the forebrain areas. The knockout and transgenic mice provide a powerful tool for investigating the roles of genes and proteins in chronic pain, but further work remains to be done. More selective signaling proteins that are involved in chronic pain still remain to be discovered. Combination of different techniques is clearly needed to elucidate the exact cellular mechanisms of chronic pain.

## Figures and Tables

**Table 1:** Tests for Studying Physiological and Pathological Pain

Physiological (Acute) Pain	Pathological (Chronic) Pain
*Thermal nociception*	*Inflammatory pain model*
Hot plate test	CFA
Tail flick test	Formalin test
Tail immersion test	Carrageenan
Thermal test (Hargreaves’ test)	
Cold plate test	*Neuropathic pain model*
	Chronic constriction injury of sciatic nerve
*Mechanical nociception*	Partial sciatic nerve ligation
Tail pinch	Spinal nerve ligation
Paw pressure test	Common peroneal nerve ligation
	Spared nerve injury model
*Visceral nociception*	Infraorbital nerve ligation
Colorectal distension	Loose ligation of sciatic nerve
Writhing test	
	*Diabetic model*
	Streptozotocin injection
	Alloxan injection
	
	*Chemotherapeutic agent-induced model*
	Paclitaxel
	Vincristine
	Suramin
	Cisplatin
	Oxaliplatin
	Carboplatin
	
	*Muscle pain model*
	Carrageenan
	Capsaicin
	Acidic saline
